# Phosphorus uptake, transport, and signaling in woody and model plants

**DOI:** 10.48130/forres-0024-0014

**Published:** 2024-05-06

**Authors:** Xingyan Fang, Deming Yang, Lichuan Deng, Yaxin Zhang, Zhiyong Lin, Jingjing Zhou, Zhichang Chen, Xiangqing Ma, Meina Guo, Zhaohua Lu, Liuyin Ma

**Affiliations:** 1 Center for Genomics, School of Future Technology, College of Forestry, Fujian Agriculture and Forestry University, Fuzhou 350002, Fujian Province, PR China; 2 Haixia Institute of Science and Technology, Fujian Agriculture and Forestry University, Fuzhou 350002, Fujian Province, PR China; 3 Research Institute of Tropical Forestry, Chinese Academy of Forestry, Guangzhou 510520, Guangdong Province, PR China; 4 College of Landscape Architecture, Guangdong Eco-engineering Polytechinic, Guangzhou 510520, Guangdong Province, PR China; 5 Fujian Academy of Forestry, Fuzhou 350012, Fujian Province, PR China; 6 College of Horticulture and Forestry Sciences, Huazhong Agricultural University, Wuhan 430070, Hubei Province, PR China; 7 State Key Laboratory of Efficient Production of Forest Resources, National Engineering Research Center of Tree Breeding and Ecological Restoration, College of Biological Sciences and Technology, Beijing Forestry University, Beijing 100083, PR China

**Keywords:** Phosphorus, Uptake, Transport, Signaling, Woody Plants

## Abstract

Phosphorus (P), a critical macronutrient for plant growth and reproduction, is primarily acquired and translocated in the form of inorganic phosphate (Pi) by roots. Pi deficiency is widespread in many natural ecosystems, including forest plantations, due to its slow movement and easy fixation in soils. Plants have evolved complex and delicate regulation mechanisms on molecular and physiological levels to cope with Pi deficiency. Over the past two decades, extensive research has been performed to decipher the underlying molecular mechanisms that regulate the Pi starvation responses (PSR) in plants. This review highlights the prospects of Pi uptake, transport, and signaling in woody plants based on the backbone of model and crop plants. In addition, this review also highlights the interactions between phosphorus and other mineral nutrients such as Nitrogen (N) and Iron (Fe). Finally, this review discusses the challenges and potential future directions of Pi research in woody plants, including characterizing the woody-specific regulatory mechanisms of Pi signaling and evaluating the regulatory roles of Pi on woody-specific traits such as wood formation and ultimately generating high Phosphorus Use Efficiency (PUE) woody plants.

## Introduction

Plant growth and development are highly dependent on the availability of soil mineral nutrients, and nutrient deficiency restricts plant productivity and reproductivity^[1,2]^. Phosphorus (P), one of the essential macronutrients, acts as a structural and functional component of nucleic acids (DNA, RNA), biomembrane phospholipids, phosphate-ester (e.g., glucose-6-phosphate), and energy-rich phosphates (ATP)^[1−3]^. Moreover, Inorganic P (Pi) is a substrate or end-product in many enzyme reactions. It regulates enzyme activity through protein phosphorylation or dephosphorylation^[3]^. Therefore, P plays a ubiquitous role in photosynthesis, respiration, energy transfer and storage, sugar metabolism, cell proliferation, cell metabolism, cell growth, and genetic information transfer^[3,4]^. In addition, P regulates many critical developmental processes, such as root development, early shoot growth, root/shoot ratio, seed formation and germination, fruit quality control, etc^[5,6]^.

P is mainly absorbed by plants in the form of inorganic orthophosphate (either H_2_PO_4_^−^ or HPO_4_^2−^) from the soil^[7]^. The concentration of inorganic orthophosphate in the soil is only between 0.1% and 0.5%^[7]^. Although plant uptake of the H_2_PO_4_^−^ form is higher than the HPO_4_^2−^ form (in the soil below pH 7.2, H_2_PO_4_^−^ > HPO_4_^2−^)^[7]^, the Pi transport within the soil is primarily by P diffusion^[7]^. However, the diffusion rate of H_2_PO_4_^−^ (0.13 mm/day) is much slower than other soil ions (e.g., NO_3_^−^, 3.0 mm/day; K^+^, 0.9 mm/day)^[7]^. Moreover, Pi precipitates with Calcium (Ca) and Magnesium (Mg) to form the Ca-P and Mg-P in neutral and calcareous soils^[7]^. Similarly, Al-P and Fe-P are the most abundant P minerals in acidic soils^[7]^. Significantly, plants cannot absorb these precipitations^[7]^. Thus, the low amount of Pi concentration, a slower Pi diffusion rate, and precipitation of Pi with cations in soils lead to continuous Pi deficiency for plant growth and development.

Pi deficiency is ubiquitous in acidic soils due to the Al-P and Fe-P oxides^[7]^. About 30% of the world's ice-top-free land areas are acidic soils^[7]^. Moreover, 52% of South America, 35% of North America, and 34% of Asia’s ice-top-free land are acidic soils^[7]^. Therefore, Pi is the second most frequent environmental factor limiting plant growth in many natural ecosystems^[8]^. Unlike agricultural production, which uses non-renewable rock phosphate as the Pi fertilizer^[2]^, forest plantations rarely use large amounts of Pi fertilizer due to the high cost. Thus, increasing the Pi content in soil from the P cycle, mobilizing the Pi from the cation-P, or developing the high P use efficiency (PUE) plants are potential strategies to alleviate the Pi starvation for forest production^[9−11]^. Currently, most woody plant P studies focus on the first two topics, with much less attention to how woody plants respond and adapt to Pi starvation from molecular aspects^[9−11]^. With the release of more woody plant genomes^[12]^, there is an urgent need to understand the molecular mechanism of how woody plants absorb, transport, and, more importantly, regulate development through Pi starvation signaling. This review will systematically prospect the uptake, transport, local Pi signaling, systematic Pi signaling, and interaction between Pi and other mineral nutrients in woody plants based on a backbone of model and crop plants.

## Effect of Pi deficiency on plant growth

In soil with a pH < 5.0, the soluble Pi reacts with Al/Fe to form Al-P/Fe-P oxides^[7]^. These oxides are insoluble and dominant in highly weathered and acidic soils^[7]^. Thus, Pi deficiency restricts the growth and development of woody plants in acidic soils.

### Efect of Pi deficiency on root system architecture

**Figure 1:**
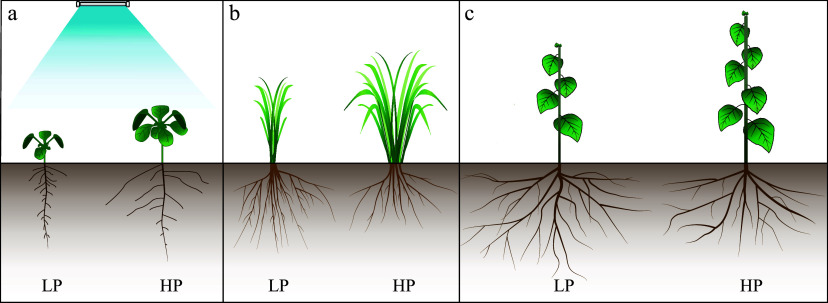
Plant phenotype response in different phosphorus statuses. Phosphorus is one of the critical macronutrients for the growth and development of plants. When phosphorus availability varies, it triggers a cascade of physiological and morphological responses. These responses are manifested in different plant phenotypes. Under phosphorus-deficient conditions (LP), plants exhibit an adaptive strategy by increasing root length, density, and branching to produce more lateral roots and longer root hairs to improve their ability to take up more phosphorus from the soil. In addition, plant growth is often stunted with reduced shoot biomass and smaller leaves, limiting photosynthesis. Stem elongation is also affected, resulting in shorter plants, and there is a tendency for plants to increase the ratio of root-to-shoot (such as LP in (a)−(c)). In contrast, when phosphorus is abundant or in high status, the root system tends to develop more normally, without the exaggerated expansion that occurs under deficiency conditions. Roots and shoots are kept in equilibrium. It will show vigorous vegetation with larger leaves, more leaves, and normal stem length (such as HP in (a)−(c)).

Pi deficiency affects the establishment of root system architecture (RSA) in plants (Fig. 1). In Arabidopsis, Pi deficiency suppresses primary root growth in a blue-light dependent manner^[5,13,14]^ (Fig. 1a). Conversely, the root cells and the roots are typically elongated under Pi deficiency in crops^[5,15]^ (Fig. 1b). However, this is not always the case, and root elongation in response to external Pi limitation depends on the genotype of a given species (e.g., soybean), of which 111 soybean genotypes exhibit different root elongation phenotypes under Pi deficiency^[16]^. Root elongation results not only from a decrease in the long-distance Pi transport of root to shoot, but also from an increase in Pi redistribution from shoot to root in *Stylosanthes hamata*^[3]^. Similar to crop plants, Pi deficiency also increases the root length of poplar^[17]^ (Fig. 1c). Low Pi also affects the Pi allocation from root to shoot, and the transport of newly acquired Pi to the oldest leaves is disrupted in *Populus* *canescens*^[18]^. However, one difference between the crop and woody plants is that newly acquired Pi is allocated to the root side of the root-shoot junction instead of actively growing tissues, including the fine root tips in *Populus* *canescens*^[18]^.

Pi deficiency also inhibits the lateral root development in Arabidopsis^[14]^ (Fig. 1a). However, Pi deficiency increases the length and density of adventitious roots in crop plants^[5,19]^ (Fig. 1b). In woody plants, Pi deficiency also stimulates the emergence and number of adventitious roots in *Populus ussuriensis*^[20]^, *Populus tomentosa* Carr^[17]^, and apple (*Malus domestica*)^[21]^ (Fig. 1c). Therefore, different with Arabidopsis, Pi deficiency induces the formation of adventitious roots in crop and woody plants to forage the Pi from the topsoil. Pi deficiency reduces the dry weight of roots in Arabidopsis, crop, and woody plants^[5,18,22]^.

Pi deficiency also induces the formation of dauciform roots and cluster roots to efficiently mine the Pi in some species from *Cyperaceae* and lupin^[23−25]^. In woody plants, Pi deficiency does not induce dauciform root formation but promotes cluster roots development in species from *Proteaceae*^[26]^. These cluster roots are critical for the survival of shrubs and trees of *Proteaceae* in the severely Pi deficiency soil of Australia and South Africa^[5]^. The cluster roots in these woody plants can reach over 40% of total root biomass and account for ~80% of new seasonal root growth under extreme Pi deficiency conditions^[5]^. Notably, cluster roots contribute to the extreme Pi deficiency stress tolerance from a higher Pi uptake efficiency and, more importantly, from extruding the organic acids to release the Pi from cation-P oxides^[27]^.

The relationship between RSA and Pi acquisition efficiency is tightly correlated in crops and ideal RSA has been screening for crops such as soybean^[28,29]^. Several studies show that soybean varieties with a shallow RSA to forage the Pi from topsoil have a higher Pi acquisition efficiency than that with a deep root^[5,29]^. Therefore, it is also urgent to understand the relationship between RSA and Pi acquisition efficiency in woody plants. What is the best RSA for woody plants? What is the RSA difference for trees with shallow and deep root systems? Similar to crops, do trees with a shallow root system also have a higher Pi acquisition efficiency? Do trees have a different strategy to mine Pi by modulating RSA than crops? These potential questions are encouraged to be addressed in the future to provide valuable information for using RSA in engineering or screening of high PUE woody plants.

### Effect of Pi deficiency on shoot growth

Pi deficiency suppresses plant shoot growth (Fig. 1). Consistent with Arabidopsis and crops^[5,30]^, Pi deficiency reduces the expansion and number of leaves in woody plants^[3]^ (Fig. 1c). However, how Pi deficiency regulates the number and size of leaves remains unexplored. One possible direction is to test whether Pi deficiency inhibits leaf development by reducing cell division zone, cell division rates, cell production, or all of these processes in woody plants. Notably, the photosynthetic rates increased by 7% after 10 d of low Pi treatment but decreased to 27% after 30 d of treatment in apple^[31]^. Moreover, Pi deficiency reduces photosynthesis by impairing the electron transport from photosystem II to photosystem I in *Citrus grandis*^[32]^. Therefore, similar to the observations from Arabidopsis and crops^[5]^, Pi deficiency suppresses leaf growth and reduces the photosynthetic rates in woody plants. In barley, impaired photosynthesis in response to Pi deficiency is a fully reversible process and can be restored within one hour after resupplying sufficient Pi^[33]^. Notably, sufficient Pi is also required for iron deficiency-mediated photosynthesis reduction, and this process is controlled by the chloroplast retrograde signaling pathway in Arabidopsis^[34]^. Therefore, it is urgent to understand whether Pi deficiency-mediated photosynthesis inhibition can be reversible in woody plants. How do woody plants balance Pi to control photosynthesis as Pi deficiency or Pi sufficient reduces photosynthesis? As some woody plants are gymnosperms, it is also interesting to test how the Pi deficiency regulates the number and size of needles in gymnosperm plants. Do needles have different responses to Pi deficiency compared to leaves? Notably, one of the crucial features of woody plants is perennial growth^[12]^. Understanding whether Pi deficiency regulates the growth of leaves/needles in a seasonal growth pattern will be interesting.

Leaf angle is an important agronomic trait, and erect-leaf rice is more suitable for dense planting to increase yield production^[35]^. Pi deficiency induces rice leaf erectness by repressing the cell elongation of lamina joint cells^[35]^ (Fig. 1b). Briefly, Pi deficiency induces SPX1 and SPX1 interaction with RLI1, preventing RLI1 from activating lamina joint cell elongation via Brassinosteroid Up-regulation 1 (BU1) and BU1-like1 complex 1, ultimately leading to erect leaf growth^[35]^. However, how the Pi deficiency affects leaf or branch angles remains unexplored in woody plants. It will be interesting to test whether leaf or branch angles are affected by Pi deficiency in woody plants to affect the efficiency of sunlight capture. Similar to crop plants, Pi deficiency also restricts tiller growth in bamboo^[36]^.

Similar to Arabidopsis and crops^[37]^, plant height is significantly reduced by Pi deficiency in *Populus* *canescens*, *Populus* *tremuloides*, apple, and Chinese fir (*Cunninghamia lanceolata*)^[18,22,31]^ (Fig. 1c). Therefore, identifying essential regulatory genes for modulating plant height is an important crop strategy^[37]^. Recently, it has been found that a transcription factor, MYB110, modulates plant height under Pi deficiency conditions in rice^[37]^. Moreover, mutation or inactivation of MYB110 leads to increased plant height, culm diameter, resistance to bending and lodging, and even grain yield under Pi deficiency conditions^[37]^. Therefore, *MYB110* is an ideal candidate gene for engineering plant height and stem diameter. Diameter at Breast Height (DBH) is the diameter of a tree trunk at a height of 1.37 m (4.5 ft) above the forest floor^[38]^. DBH is used to estimate the total volume and biomass of trees^[38]^. Therefore, the height and DBH are two critical factors to evaluate the growth and productivity of forest trees^[39]^. If a tree grows tall and has a thick DBH, this represents the maximization of wood production, which is the ideal goal of wood improvement. Thus, it is urgent to understand whether Pi deficiency simultaneously regulates the plant height and DBH. Do woody plants have master regulatory genes simultaneously regulating plant height and DBH? What is the underlying molecular mechanism of Pi deficiency-mediated plant height reduction in woody plants?

Wood is an essential component of shoot biomass, and wood formation is the most interesting feature of woody plants^[40]^. However, the function of Pi in the regulation of wood formation has not been characterized so far. Although studies in woody plants show that the concentration of P does not accumulate in wood^[41]^, it cannot be excluded that Pi regulates wood formation *via* interplaying between P and other nutrients. Notably, Potassium (K), Ca, and Boron (B) are known to be required for wood formation, and deficiency of these nutrients greatly affects the wood formation traits such as cambial activity^[42,43]^. As Pi interacts with K, Ca, and B^[38−41]^, it will be interesting to study whether Pi regulates wood formation directly or indirectly through interactions with other nutrients in woody plants.

Similar to Arabidopsis and crops^[3]^, Pi deficiency suppresses the dry and fresh weight of woody plants such as *Populus canescens*^[15]^, *Populus tremuloides*^[15]^, eucalyptus (*Eucalyptus grandis*)^[42]^, and apple^[31]^. Therefore, Pi deficiency restricts shoot growth in woody plants. A schematic diagram is drawn to compare the effects of phosphorus deficiency on growth and mycorrhizal fungal symbiosis in Arabidopsis, rice, and poplar (Table 1).

**Table 1:** The effects of phosphorus deficiency on growth and mycorrhizal fungal symbiosis in Arabidopsis, rice, and poplar.

Species	Root				Shoot							Mycorrhizal symbiosis	
	Primary root	Lateral root	Shoot/root ratio		Leaf number	Leaf growth	Leaf angle	Height	Photosynthetic	Shoot biomass		AM	ECM
Arabidopsis	Inhibit	Inhibit	Reduce		Reduce	Inhibit	–	Reduce	Inhibit	Reduce		No	No
Rice	Enhance	Enhance	Reduce		Reduce	Inhibit	Reduce	Reduce	Inhibit	Reduce		Colonize	No
Poplar	Enhance	Enhance	Reduce		Reduce	Inhibit	–	Reduce	Inhibit	Reduce		Colonize	Colonize
AM: Arbuscular Mycorrhizal fungi; EM: Ectomycorrhizal fungi; '–' represents unexplored.													

## Pi uptake and transport

Plants have developed sophisticated transport systems to absorb the Pi from soil to root cells, transport the Pi from root cells to the subcellular organelles (e.g., vacuole), or translocation from root to other tissues or organs (e.g., root to shoot)^[2,5]^.

### Pi uptake in roots

**Figure 2:**
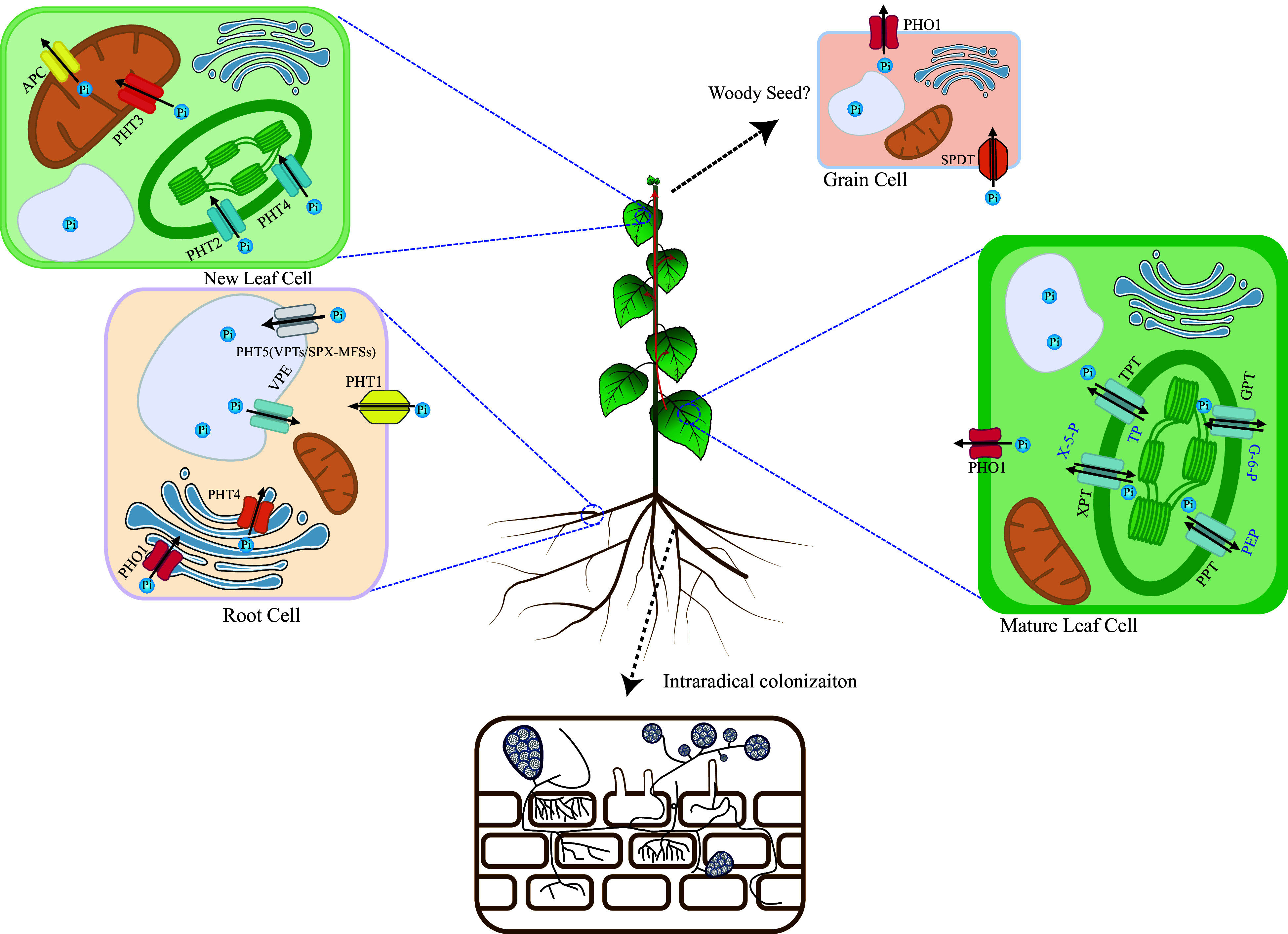
Pi uptake, transport, and remobilization transporters in plants. In plants, several types of transporters are involved in the complex uptake, transport, and remobilization of inorganic phosphate (Pi), the primary form of phosphorus taken up by roots. Intracellular Transport: At the root-soil interface, Pi is taken up from the soil solution by high-affinity transporters, mainly PHT1 family proteins. Once inside the cytoplasm, Pi is a central hub for exchanging Pi between different organelles. Several transporters shuttle Pi across organellar membranes. For example, PHT/VPE transporters can move Pi into vacuoles for storage or release it when needed. Other transporters manage Pi fluxes into and out of chloroplasts (TPT, PHT2, PHT4, XPT, PPT, GPT), mitochondria (PHT3, APC), and the Golgi (PHT4, PHO1) apparatus to support essential metabolic processes. Long-Distance Transport: The long-distance transport of inorganic phosphate (Pi) by the PHO1 transporter, primarily localizing at cellular membranes, is a critical process that ensures the efficient distribution of this essential nutrient from roots to shoots and the developing tissues. PHO1 facilitates the loading of Pi into the xylem sap for its systemic movement through the vascular system of the plant. Pi Remobilization in Mature Leaves: During senescence or phosphorus deficiency, Pi can be remobilized in mature leaves. PHO1 and possibly PHT1 transporters help to mobilize stored or excess Pi back into the cytoplasm. This prepares it for export to younger leaves or developing grains. Pi transport in Grains: Developing grains requires significant Pi to grow and mature. Specific Pi transporters, such as SPDT, can mediate the uptake of external Pi into grain cells. And PHO1 plays a role in expelling Pi from grains when necessary.

To cope with the low concentration of Pi in soils, plants develop an efficient transport system to absorb Pi directly from soils and transport them into root cells (e.g., rhizodermal cells)^[2]^. Phosphate Transport 1 (PHT1), a group of plasma membrane-localized transporters, acts as the only reported Pi influx transporters in Arabidopsis, crops, and woody plants^[44,45]^ (Fig. 2). The *PHT1* gene family has multiple members and numerous *PHT1s* have been identified in woody plants^[8,9]^. To date, 12, 22, 14, seven, six, 23, and 12 *PHT1s* have been characterized in poplar^[8]^, eucalyptus^[46]^, apple^[47]^, trifoliate orange (*Poncirus trifoliata* L. Raf.)^[48]^, wolfberry (*Lycium barbarum* L.)^[49]^, tea plants (*Camellia sinensis* L. O. kuntze)^[50]^, and rubber tree (*Hevea brasiliensis*)^[51]^, respectively.

Consistent with Arabidopsis and rice^[2]^, gene expression analyses unveil that *PHT1*s are mainly expressed in the roots of woody plants. For example, eight of 12 *PHT1*s are highly expressed in poplar adventitious roots^[8]^. At least nine of 14 *PHT1s* are expressed in apple roots^[47]^. Eleven of 12 *PHT1s* are expressed in rubber tree roots^[51]^. Notably, the maximum Pi uptake rate is 13 times higher in Pi deficiency poplar than in Pi sufficient poplar^[18]^. Pi deficiency induces the expression of most *PHT1*s in poplar^[8]^, apple^[47]^, trifoliate orange^[48]^, and rubber tree^[51]^. Therefore, these results indicate that PHT1-associated Pi uptake is critical for Pi deficiency tolerance in woody plants.

The function of several woody PHT1s in Pi deficiency tolerance is also validated by successfully complementing woody PHT1 into the yeast (*Saccharomyces cerevisiae*) Pi transporter mutant strain EY917 (Δnull) under Pi deficiency conditions^[46,51]^. One of the important strategies to cope with Pi deficiency is to identify the functional PHTs under Pi deficiency. However, most woody plants have over ten members of PHT1s^[8,42,45,48,49]^, and functional analyses of each woody PHT1s remain largely unexplored. More studies are expected to be performed within woody plants by genetic over-expression and mutation by CRISPR-Cas9 or RNAi to identify the key Pi deficiency associated PHT1s in each woody plant. These functional PHT1s will be ideal candidates to generate high PUE woody plants by transgenic or CRISPR-Cas9 technology in the future.

### Intracellular compartmentation of Pi

About ~85%−90% of total Pi is stored in the vacuole as storage pool under sufficient Pi supply but exported to cytosol and chloroplasts under Pi deficiency condition^[3]^. Therefore, the tonoplast transporters of Pi act as the ON and OFF switches for Pi storage and activation in cells. Vacuole Phosphate Transport 1 (AtVPT1)/AtPHT5;1, AtVPT3, OsSPX-MFS1, OsSPX-MFS2, and OsSPX-MFS3 are Pi influx, while Vacuolar Pi Efflux transporters (OsVPE1 and OsVPE2) have been identified as Pi efflux in Arabidopsis and rice^[52−55]^ (Fig. 2).

Photosynthesis provides energy for plant survival, and sufficient Pi in chloroplast is critical for photosynthesis^[33]^. TPT, PHT2;1, and PHT4;4 are chloroplast Pi influx in Arabidopsis and rice^[56−59]^ (Fig. 2). Notably, TPT is a 3-phosphoglycerate or triose-phosphate/Pi antiporter to modulate the chloroplast Pi homeostasis and accounting for 10%−12% of chloroplast inner envelop membranes localized proteins^[56,57,60]^. Similarly, PHT2;1 is characterized as a low-affinity chloroplast Pi influx in Arabidopsis and rice^[58,59]^, while PHT4;4 is assumed as a chloroplast Pi influx in Arabidopsis^[61]^. Conversely, Phosphoenolpyruvate/Phosphate Translocator (PPT), Xylulose 5-phosphate/Phosphate Translocator (XPT), and Glucose 6-phosphate/Phosphate antiporter (GPT) are chloroplast Pi efflux in Arabidopsis^[62−64]^ (Fig. 2).

Sufficient Pi is critical for the oxidative phosphorylation of ADP to ATP in mitochondria^[65]^. PHT3/MPT act as the mitochondria Pi influx by Pi/H^+^ symporter and Pi/OH^−^ antiporter in Arabidopsis^[65]^ (Fig. 2). Conversely, ATP/Phosphate Carriers (APCs) are characterized as mitochondria Pi efflux in Arabidopsis^[66]^ (Fig. 2). According to localization analyses, Phosphate 1 (PHO1) is predicted to act as the Pi influx of Golgi in Arabidopsis^[67]^, while PHT4;6 is the Pi efflux of Golgi to recycle Pi from nucleotide-diphosphate sugars that are used for protein glycosylation in Golgi in Arabidopsis^[68]^ (Fig. 2). However, functional validation of PHO1 and PHT4;6 in Golgi Pi homeostasis remains largely elusive even in model plants.

The tonoplast, chloroplast, mitochondria, and Golgi Pi transporters in woody plants remain largely unexplored. Notably, PHT2s, PHT3s, and PHT4s have been characterized in poplar^[18]^, apple^[47]^, and tea plants^[50]^, but their function on intracellular organelles Pi homeostasis remains elusive. Identifying and characterizing the Pi transporters localized in different intracellular organelles is urgent. One interesting question that can be evaluated is how woody plants balance Pi between storage and activation organelles. More importantly, how woody plants modulate intracellular Pi homeostasis during wood-specific processes such as wood formation and seasonal growth. Is there any difference between the gymnosperm and angiosperm on the regulatory mechanisms of Pi homeostasis? Therefore, it is important to characterize these intracellular organelles Pi transporters and unveil woody-specific intracellular Pi homeostasis in the future.

### Long-distance Pi transport

Pi is transferred from the root cell into the xylem vessel through the Casparian band by PHO1s, the long-distance Pi transporter from root to shoot^[69]^ (Fig. 2). Although ten and three PHO1 are characterized in Arabidopsis and rice respectively, only two Arabidopsis PHO1s (AtPHO1 and AtPHO1;H1) and one rice PHO1 (OsPHO1;2) functions in exporting Pi from root to xylem^[70,71]^. Although 12 PHO1s have been characterized in poplar^[72]^, the function of these PHO1s on long-distance transport has not been evaluated. However, gene expression analyses indeed show an expression pattern difference between Arabidopsis and poplar PHO1s under Pi deficiency conditions. Three of the Arabidopsis PHO1s (AtPHO1, AtPHO1;H1, and AtPHO1;H10) are low Pi-induced genes^[70]^, while Pi deficiency cannot alter the mRNA level of poplar PHO1s^[72]^. One of the possible directions for studying the long-distance Pi transporter is to understand whether there is any difference at the molecular level between the gymnosperm tracheid and angiosperm vessel systems in woody plants.

### Remobilization of Pi from mature leaves

Pi is mobile and can be redistributed across different tissues^[2,73]^. Similar to model plants, it has been shown that leaves, not wood, provide the primary sources of Pi for internal remobilization in the evergreen oak (*Quercus ilex*)^[41]^. Therefore, vacuoles of mature or senescing leaves are primary Pi sources in woody and other plants. Pi can transfer from mature or senescing leaves (source) to sink tissues, including young leaves and newly developing seeds in model and crop plants^[2,73]^. OsPHT1;7, OsPHT1;8, and its maize homolog-ZmPT7 function on the redistribution of Pi from mature leaves (source) to young leaves (sink)^[45,74,75]^. Notably, PHO1;1 is expressed in the rice companion and xylem parenchyma cells to modulate the Pi transporting from the leaf tip to the leaf base^[76]^. However, the Pi remobilization transporters have not been characterized in woody plants.

### Pi transport in grains

SULTR-like Phosphorus Distribution Transporter (SPDT) is a xylem-localized transporter that allocates Pi from leaves to grain in rice, barley, and Arabidopsis^[77]^ (Fig. 2). However, barley SPDT contributed more to grain Pi accumulation than rice SPDT^[78]^. This is because barley SPDT is expressed in both the xylem of enlarged vascular bundles and the phloem of diffuse vascular bundles and plays a dual role in Pi transport, unloading Pi in the xylem and reloading Pi in the phloem; however, rice SPDT is expressed only in the xylem parenchyma cells, and thus acts to unload Pi only in the xylem of enlarged vascular bundles^[78]^. In rice, OsPHO1:1 is also required to reload Pi into the phloem of diffuse vascular bundles^[79]^. Arabidopsis SPDT also transports Pi from mature to new leaves^[80]^. Thus, the functions of SPDT are diverse in different species. However, SPDT has not been characterized in woody plants. It will be interesting to evaluate whether SPDT in woody plants has a different role than its rice and barley homologs. Conversely, the PHO1 transports the Pi from grains to other tissues^[79,81]^ (Fig. 2). Similar to SPDT, the role of PHO1 in the efflux of Pi from the grain in woody plants has not been characterized.

### Pi uptake and mycorrhizal symbiosis

Plants and mycorrhizal fungi are mutually beneficial through mycorrhizal symbiosis: the mycorrhizal fungi provide the plants with water and mineral nutrients such as phosphorus and nitrogen in exchange for fatty acids and sugars from plants^[82,83]^ (Fig. 2). Arbuscular Mycorrhizal (AM) fungi, Ectomycorrhizal (ECM) fungi, Ericoid Mycorrhizal (ERM) fungi, and Orchid mycorrhizal (ORM) fungi are the four main mycorrhizal fungi, according to their functions and structures^[82]^. AM symbiosis is the most dominant mycorrhizal symbiosis, and ~70%−90% of land plants form AM symbiosis with the only fungi from *Glomeromycota*^[82]^. The ECM fungi host plants almost all belong to woody plants (*Salicaceae,* *Pinaceae*, *Fagaceae*, *Betulaceae*, and *Dipterocarpaceae*)^[82]^. Unlike AM fungi, ECM fungi are highly diverse^[82]^. ERM fungi are host-specific for symbiosis only in the *Ericaceae* (Heath family), consisting mainly of shrubs and small trees^[82]^. The ORM fungi are host-specific and only colonize with the *Orchidaceae* family, the largest monocotyledonous plant family^[82]^. Therefore, woody plants can establish symbiosis with AM, ECM, and ERM fungi to enhance mineral nutrient absorption. Moreover, many woody plants can inoculate with more than one type of mycorrhizal fungi; for example, plants from *Acacia*, *Eucalyptus*, *Populus*, *Alnus*, *Fraxinus*, *Shorea*, *Salix*, and *Uapaca* are dual-mycorrhizal plants, and they can form symbiosis with both AM and ECM fungi^[84]^. Notably, ECM symbiosis is more abundant in temperature forests, while AM symbiosis is more dominant in the subtropic and tropic forests^[82]^.

Mycorrhizal fungi enhance phosphate uptake by symbiotic plants in several ways. One is accessing phosphate outside the rhizosphere through their extraradical hyphae, which also release organic acids that dissociate inorganic phosphorus from its fixed oxides (Fe-P, Ca-P)^[82]^. Another is the secretion of extracellular phosphatases (mostly by ECM mycorrhizal fungi), which release Pi from organic sources such as nucleic acids, phospholipids, and proteins^[82]^. In addition, mycorrhizal fungi interact with phosphate-solubilizing bacteria (PSB) to synergistically dissolve insoluble Po into soluble Pi^[82]^.

Phosphate transporters localized in the external hyphae of AM fungi are responsible for the uptake of Pi from the environment^[82]^. These Pi subsequently form polyphosphates (poly-P) and undergo several translocations to reach the arbuscular branches, where they are hydrolyzed back to Pi^[82]^. The plant root system then takes up these Pi *via* PHTs located in periapical membranes, and the AM symbiosis induces the expression of *PHTs*^[82]^. Similarly, the ECM has specialized PHT transporters that take up Pi through ectomycorrhizal hyphae^[82]^. These Pi form poly-P, most of which are stored in the fungal hyphae^[82]^. However, some poly-P from ECM is translocated into the symbiotic apoplastic space and finally into plant cells *via* the plant's PHT transporter^[82]^. Thus, AM and ECM symbiosis induces the expression of plant *PHT1s*. The expression of many woody *PHT1s* expression is increased after inoculating AM and is being proven to be closely associated with AM-directed Pi uptake under Pi deficiency in poplar^[8]^, eucalyptus^[46]^, trifoliate orange (*Poncirus trifoliata* L. Raf.)^[48]^, and wolfberry (*Lycium barbarum* L.)^[49]^. Similarly, the expression of many woody PHTs is also enhanced by ECM under Pi deficiency in *Pinus sylvestris*^[85]^, masson pine (*Pinus massoniana*)^[86]^, poplar^[8]^, and jarrah (*Eucalyptus marginata*)^[87]^. Moreover, both AM and ECM induce the expression of two *PHT*s from poplar (*Populus trichocarpa*)-*PtPT9* and *PtPT12*^[8]^. In addition, ERM also induces the expression of phosphate transporters from blueberry (*Vaccinium* *spp.*)^[88]^ and *Rhododendron fortune*^[89]^. Therefore, AM, ECM, and ERM fungi enhance Pi absorption by up-regulating the expression of *PHTs* in woody plants.

Therefore, screening the high PUE-associated AMs and developing the bio-fertilizer for each economic woody plant is expected to improve high PUE in woody plants. In addition, understanding how AM and ECM fungi interact to affect Pi uptake in woody plants is an interesting question to be answered in the future. Recently, the Pi uptake and mycorrhizal symbiosis have been reported to be regulated by the Pi signaling core transcription factor, Phosphate Starvation Response 2 (PHR2) in rice^[83]^. It will be interesting to test how the Pi uptake, Pi signaling, and AM symbiosis regulate each other in woody plants by integrating the Y1H-seq, Y2H-seq, and different genetics and molecular methods^[90]^.

### Strategies for the study of Pi transporters in woody plants

Three potential strategies may be adopted to characterize the Pi transporters and decipher their function in woody plants. The first and most common strategy is functional analyses of woody Pi candidate transporters by complementing experiments^[46]^. The idea is to either complement these woody Pi candidate transporters encoding genes into yeast transporter mutants to evaluate their transport activity or complement them into the transporter mutant of model plants^[46]^. In this way, one can prove the function of these woody Pi transporters. However, this strategy is limited because novel or woody-specific Pi transporters cannot be established due to a lack of yeast or model plant mutants.

Another strategy adopted in model plants is coupling genetics and ionomics profiling to identify novel transporters in model plants^[91]^. For example, once a gene is mutated by either forward genetics or natural variation, if it is a mineral nutrient transporter, the ionomics profiling detected by inductively coupled plasma mass spectrometry (ICP-MS) is changed^[91]^. In this way, many mineral transporters are characterized in Arabidopsis^[91]^. Therefore, combining genetics and ionomics profiling are potentially useful techniques for characterizing the mineral nutrient transporter in woody plants.

Recently, a newly developed Pi cell visualization technique, inorganic orthophosphate staining assay (IOSA), has been reported^[76]^. IOSA can generate a semi-quantitively high-resolution image to show how a mutant Pi candidate transporter can affect intracellular Pi homeostasis^[76]^. This method has been successfully applied and proved by several well-known Pi transporters in Arabidopsis and rice^[76]^. Notably, OsPHO1;1, a novel Pi redistribution transporter in rice leaf, has been successfully identified by this technique^[76]^. By introducing these techniques, it is expected that there will be more and more Pi transporters being characterized in the woody plants.

## Pi starvation signaling

Due to the inherent non-uniformity and poor mobility of Pi in soils, plants have evolved a sophisticated PSR that detects and integrates Pi concentration levels^[1,2]^. When Pi is deficient, the energy-consuming PSR pathway triggers enhanced root growth and development for more efficient phosphorus acquisition from soils. Conversely, when Pi is enriched, the PSR system promptly suppresses further activation, thus conserving valuable energy resources^[1]^. Extensive research using root-splitting experiments and transcriptional profiling has shown that plants have two distinct categories of PSR. The first type is local Pi signaling, controlled by the immediate, localized phosphorus concentration at the root-soil interface. The second type is systemic Pi signaling, which reflects the overall or global Pi status within the plant and involves long-distance signaling pathways that coordinate the plant response across different organs^[1,92,93]^.

### Local Pi signaling

In response to local Pi starvation, plants' RSA undergoes significant changes. Typically, this involves a suppression of primary root growth, an enhancement of lateral root development, and an increase in root hair density and elongation^[2,94,95]^. These RSA changes are primarily influenced by external soil Pi concentrations rather than internal plant Pi levels^[2,96]^. The primary root growth arrest is the most prominent RSA phenotype under PSR driven by local Pi signaling in Arabidopsis^[1,97,98]^.

Pi deficiency-induced transcriptional reprogramming affects approximately 20% of the plant transcriptome^[99]^. Transcription factors (TFs) play a central role as key regulators of these processes, in which Sensitive To Proton rhizotoxicity 1 (STOP1) is central to local Pi signaling^[1]^. STOP1, a C_2_H_2_ Zinc-finger family transcription factor, is known for its critical function in Al toxicity and low pH tolerance in acidic soils^[100]^. Recently, studies have shown that STOP1 regulates RSA by coordinating with another transcription factor, TCP20, to activate the expression NRT1.1 in lateral root primordia during nitrogen deficiency^[101]^.

**Figure 3:**
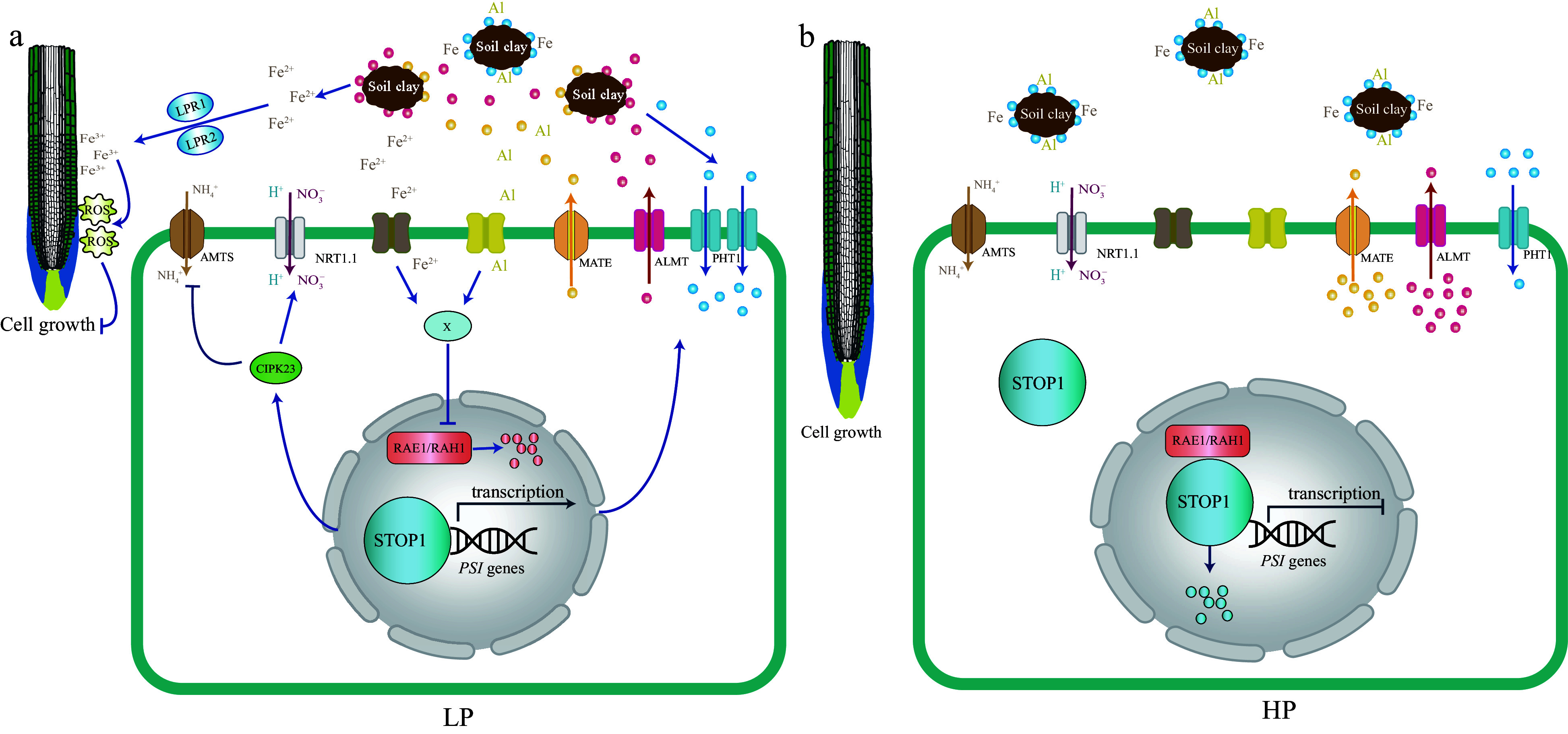
A model based on the current understanding of the local Pi signaling pathway. STOP1 acts as a master regulator integrating multiple facets of local Pi signaling, including ion homeostasis, nutrient mobilization, and tolerance to environmental stressors such as pH changes and toxic ions, particularly Iron (Fe) and Aluminum (Al). Under low Pi conditions (LP), STOP1 is primarily activated by Fe. Fe acts similarly to Al under moderately acidic conditions. It is hypothesized that extracellular Fe (or Al) may induce the accumulation of an unknown compound X in the cell, preventing the degradation of STOP1, which is controlled by the F-box proteins RAE1 and RAH1. The STOP1 activity is upregulated, which induces the expression of ALMT1, an aluminum-activated malate transporter, releasing malate into the rhizosphere. This exuded malate interacts with the ferroxidases LPR1 and LPR2 and promotes the aggregation of Fe^3+^ in the apoplast of root cells, which stimulates the formation of reactive oxygen species (ROS). The accumulation of ROS negatively impacts primary root growth by preventing elongation. In addition, ammonium transporters of the AMT1 family (AMTs in the figure), which are up-regulated by low Pi, are involved in local Pi signaling. Ammonium uptake *via* AMTs involves proton extrusion, which acidifies the rhizosphere. High rhizosphere acidity is sufficient to induce STOP1 activation, but Fe greatly enhances the level of STOP1 activation. STOP1 prevents ammonium toxicity by upregulating CIPK23 and then inhibiting AMT transporter activity. Furthermore, STOP1 directly upregulates the NRT1.1, which co-transport nitrate and protons, providing a mechanism to increase rhizosphere tolerance to low pH. Under Pi-sufficient conditions (HP), root growth is generally normal because the excess phosphate provides an adequate supply for essential cellular processes. Excess phosphorus may interact with metal ions such as Aluminum (Al) and Iron (Fe), forming complexes that make these metals less bioavailable to plants. Blue dot: Pi, Red dot: malate, Yellow dot: citrate.

A model has been illustrated to depict the current understanding of local Pi signaling of STOP1 in response to PSR (Fig. 3). Under Pi deficiency conditions, STOP1 is activated and upregulates the expression of organic acid transporters Aluminum-activated Malate Transporter 1 (ALMT1) and Multi-drug and Toxic compound Extrusion (MATE), thus increasing the exudation of malate and citrate into the soil, which not only released Pi from Al-P and Fe-P oxides, increasing Pi availability to the plant^[97,98,102]^ but also producing free Fe^2+^^[98]^. Then, the ferroxidase Low Phosphate Root 1 (LPR1) oxidizes Fe^2+^ to Fe^3+^, and malate-dependent Fe^3+^ is accumulated in the apoplast of cell elongation and meristem regions^[98,103]^, leading to robust ROS production in the apical regions of primary roots, ultimately stiffening the cell wall and inhibiting primary root growth^[98,103]^. Under Pi deficiency and low intracellular ammonium levels, the ammonium transporter AMT1 imports NH_4_^+^ into cells, activates the STOP1 signaling pathway *via* a currently unknown mechanism, and induces the nuclear accumulation of STOP1^[104]^. The NH_4_^+^ uptake also increases the proton extrusion into the rhizosphere and acidifies it^[104]^. Under high ammonium conditions, STOP1 activates the transcription of the post-translational modulator CBL-interacting Protein Kinase 23 (CIPK23), and CIPK23 subsequently represses AMT1 transport, blocking the NH_4_^+^ uptake and alleviating cellular NH_4_^+^ toxicity^[104,105]^. In addition, STOP1 can activate the Nitrate Transporter 1.1 (NRT1.1) to transport the H^+^ and NO_3_^−^ into the cells, thereby increasing tolerance to low pH in the rhizosphere^[106]^. STOP1 stability is regulated by ubiquitination and subsequent degradation by the RNA export factor 1 (RAE1) and RAE1 homolog 1 (RAH1) F-box protein-associated proteasome^[107,108]^. Although it is not yet clear, it is speculated that an uncharacterized X protein can either sense free Fe^2+^ and Al^3+^ at the plasma membrane or directly enter the cells to inhibit the proteasome of STOP1, thereby activating the STOP1 in local Pi signaling pathway^[1]^.

Conversely, under conditions of sufficient Pi availability, Pi combines with Al^3+^ and Fe^2+^ to form insoluble Al-P and Fe-P oxides^[1]^. This leads to the disruption of STOP1 activation signaling by the hypothetical X protein, and the proteasomal degradation pathways RAE1 and RAH1 are activated, leading to the degradation of STOP1^[107]^. Thus, STOP1-dependent activation and transport of organic acids is inhibited, preventing Fe^3+^ accumulation in the cell wall of the primary root^[103]^. Together, these events lead to the cell wall loosening and promote primary root growth.

In woody plants, the function of STOP1 in the aluminum tolerance has been characterized in apples and eucalyptus^[109,110]^. CIPK23 has been implicated in the low potassium stress responses in poplar and apple^[111,112]^. Although two ALMT and MATE transporters, PoptrALMT10 and PoptrMATE54, have been identified and functionally analyzed in poplar, these genes only respond to aluminum toxicity, not Pi deficiency^[113]^. AMT1 has also been depicted in poplar and tea, but its functions have primarily been investigated in the context of nitrogen deficiency^[114,115]^. The homologous proteins of LPR1, RAE1, and RAH1 in woody plants remain elusive, and thus, the roles of STOP1-related local Pi signaling pathways are largely unexplored in woody plants.

### Systematic Pi signaling

Using foliar Pi application assays and root-splitting experiments, Pi was shown to act as a systemic signal within plants^[116]^. This conclusion is further supported by the fact that the non-metabolic analog of Pi, Phosphite (Phi), can effectively inhibit the PSR of plants^[117]^. Systematic Pi signaling differs from local Pi signaling, which senses and regulates the external Pi status. This complex regulatory pathway integrates multiple processes, such as the transport, recovery, and recycling internal Pi in different plant parts. It is intrinsically linked to maintaining internal plant phosphate homeostasis^[118]^.

Like local Pi signaling, Arabidopsis PHR1 or rice PHR2 a GARP transcription factor, plays a central regulatory role within systematic Pi signaling pathways^[119]^. The function of PHR1 and its homologs PHL (PHR1-like) TFs have been extensively studied in model organisms such as Arabidopsis and economically important crops such as rice, which they have been found to significantly influence the plant response to phosphate starvation^[1]^. AtPHR1 or OsPHR2 have a strong affinity for binding to a specific PHR1 binding sequence (*P1BS*, GNATATNC)^[119−121]^. Transcriptome-wide studies have shown that when both PHR1 and PHL1 are mutated in Arabidopsis, there is a profound reprogramming of gene expression; over 2,000 genes that are typically up-regulated under Pi starvation conditions show a decrease of more than 70%, while approximately 1,800 down-regulated genes show an increase of more than 50%^[122]^. This indicates a significant impact on the transcriptional response of the plant to Pi availability. Metabolomics analyses further support this importance, showing that approximately 75% of the metabolites associated with Pi starvation in Arabidopsis^[123]^. PHR1 and PHLs regulate almost all physiological aspects of systematic PSR^[1]^. Indeed, the presence and functional role of PHR1 homologs in maintaining cellular Pi homeostasis have been documented across numerous plant species beyond Arabidopsis and rice, suggesting that PHR proteins have a universal and predominant role in regulating PSRs. Meanwhile, the PHR homology has not been found in woody plants. However, it is worth noting that although PHR1 and PHLs play a key role in systemic Pi signaling regulation, they have limited impacts on local Pi signaling^[1]^.

**Figure 4:**
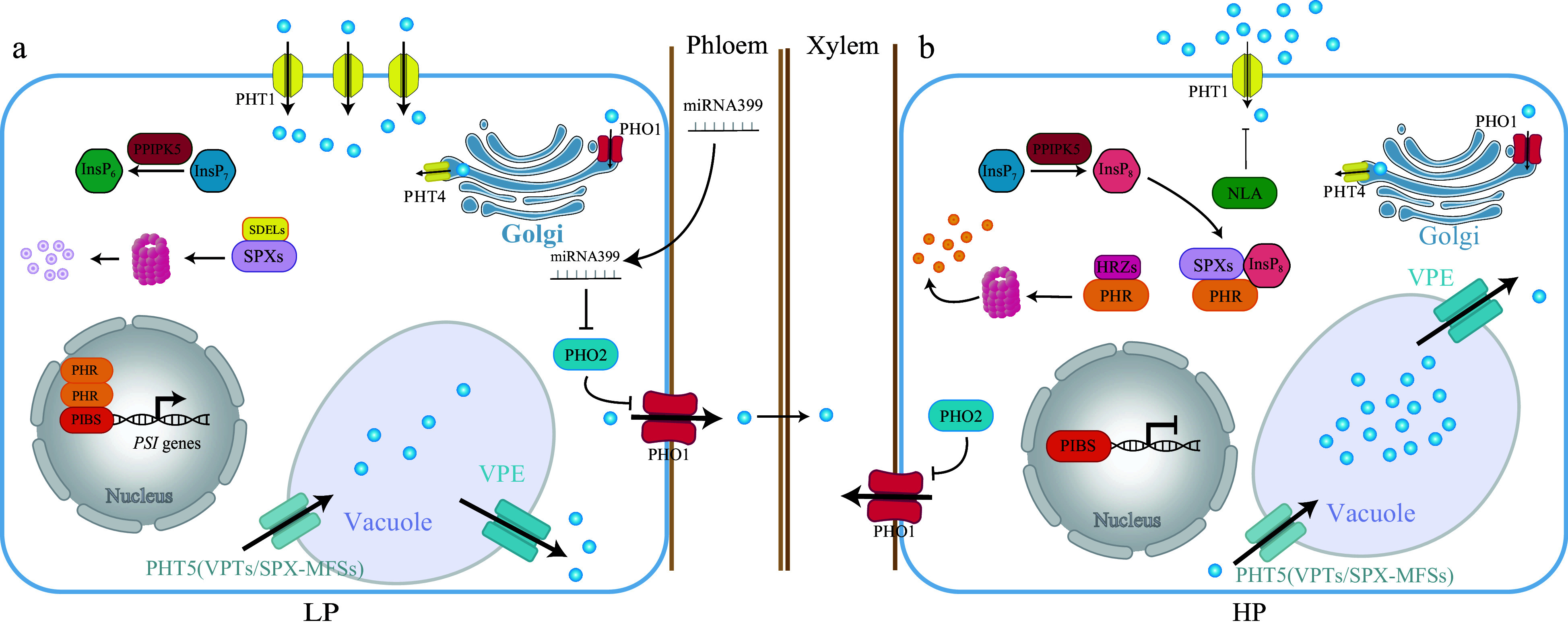
A model depicts the InsPs-SPXs-PHRs-centered systematic Pi signaling in response to Pi deficiency. Under Pi deficiency, the Pi and ATP levels are lower, and PPIP5K converts the InsP_7_ to InsP_6_, leading to a decrease in the InsP_8_ level. The SPXs-PHRs dimer is dissociated, and SPXs are degraded by SDELs^[127]^. Thus, PHRs are free to activate the transcription of *PSI* genes (*PHT1s*, *microRNA827*, *microRNA399* and *PHT5*). PHRs activate the Pi uptake by up-regulating the transcription of PHT1 transporters such as *PHT1;1*^[142]^. PHRs further increase the plasma-membrane localized PHT1;1 by increasing the transcription of *microRNA827* (*miR827*), which targets and degrades NLA, a ubiquitin E3 ligase of PHT1^[143]^. PHRs reduce the Pi storage in the vacuole by increasing the transcription of *miR827*, which also targets and degrades PHT5, a vacuole Pi influx^[53]^. PHRs stimulate the expression of the vacuole Pi efflux VPE via the *P1BS* motif^[54]^. Thus, PHRs reduce the Pi storage and increase Pi activation by increasing the expression of Pi influx but reducing the expression of Pi efflux in the vacuole, the Pi storage organelle. PHR1 also activates the transcription of *microRNA399* (*miR399*), and *miR399* moves from shoot to root upon Pi deficiency to target and degrade the mRNA of a ubiquitin-conjugating E2 genes-PHO2^[144−148]^. PHO2 degrades the PHO1 and disrupts the xylem loading of Pi from root to shoot^[148]^. Therefore, PHR1 activates *miR399* to repress PHO2 and thus ultimately leads to activating the PHO1-mediated Pi allocation from root to shoot upon Pi deficiency. Conversely, under Pi-sufficient conditions, the PPIP5K converts the InsP_7_ to InsP_8_, and InsP_8_-SPXs block the activity of PHRs. The PHRs are also degraded by HRZs^[149]^, and *PSI* genes are less activated by PHRs. The intracellular Pi is more likely to be stored in a vacuole, and Pi uptake is also reduced, ultimately reducing Pi activation by increasing the Pi storage. Thus, the PHR-centered systematic Pi signaling is not activated.

The transcriptional expression of PHRs is not significantly induced by low Pi treatment in land plants^[119]^. However, the activity of PHR is regulated by a class of SPX proteins that contain a single SPX domain^[124]^. SPX proteins act as negative regulators of the Pi starvation signaling pathway and are critical for the modulation of the Pi starvation response and maintaining Pi homeostasis^[125−127]^. SPX is proposed to act as a sensor of Pi status by binding to the important eukaryotic signaling molecule inositol polyphosphates (InsPs) and negatively regulating the function of PHR1^[124,125,128]^. In Arabidopsis, SPX1 and SPX2 are nuclear proteins interacting with PHR1 in the nucleus to prevent binding to the *P1BS* element of phosphate starvation-induced (*PSI*) genes^[124,129]^ (Fig. 4). The same mechanism is conserved in rice, where OsSPX1/OsSPX2 interacts with OsPHR2 to repress its function^[125]^ (Fig. 4). SPX proteins are induced by Pi starvation, except for SPX4^[130]^. Arabidopsis SPX4 and its rice homologs, OsSPX4 and OsSPX6, bind to PHR proteins outside the nucleus and thus restrict their translocation into the nucleus under conditions of high P availability^[124,126,131]^ (Fig. 4b). When P becomes scarce, these SPX proteins undergo ubiquitin-mediated proteasomal degradation, releasing the inhibition on PHR2 and allowing it to enter the nucleus and initiate the transcriptional response necessary for plants to cope with P deficiency^[127]^ (Fig. 4a).

SPX proteins often contain additional functional domains involved in phosphate starvation signaling in plants; for instance, PHO1 contains a transmembrane domain (EXS), is involved in xylem Pi loading; SPX-MSF contains an MSF domain associated with membrane transport, is a vacuolar Pi influx transporter; NLA contains a zinc finger domain (RING) with E3 ubiquitin ligases mediating PHT1 ubiquitination together with PHO2^[132,133]^. It is initially suggested that SPXs might be Pi sensors^[124,125]^. However, the crystal structure analysis of SPX domains shows that InsPs have a high-affinity binding activity than to the Pi (> 10,000-fold), and other genetic evidence unveils that instead of Pi, InsP_8_ are intracellular Pi sensors^[128,134,135]^. A bifunctional diphosphoinositol pentakisphosphate kinase, PPIP5K, tightly regulates the level of InsP_8_ in a Pi- and ATP-dependent manner^[128,135]^. Briefly, PPIP5K can convert the InsP_7_ either to InsP_6_ or InsP_8_^[135]^. Under Pi deficiency, the Pi and ATP levels are lower, and PPIP5K converts the InsP_7_ to InsP_6_^[135]^ (Fig. 4a). Conversely, PPIP5K converts the InsP_7_ to InsP_8_ by consuming ATP upon Pi-sufficient conditions^[135]^ (Fig. 4b). Therefore, the Pi levels can be transformed as the level of InsP_8_. The crystal structure analysis has deciphered the interaction of SPX with InsP_8_, and InsP_8_ can stabilize the N-terminal of SPX1 and result in a conformation change of SPXs-PHRs dimer to block the transcriptional activity of PHRs by SPXs^[136]^. Therefore, InsP_8_-SPXs is a negative regulatory module of PHRs.

The PPIP5K and SPXs have not been characterized and functionally analyzed in woody plants. However, the function of PHR1/PHL is involved in the PSR of woody plants^[17,137]^. For example, over-expression of MdPHR1 enhances the Pi deficiency tolerance by activating the expression of purple acid phosphatase in apples^[137]^. A PtoWRKY40-PtoPHR1-LIKE3 (PtoPHL3)-PtoPHT1 regulatory module for PSR has been identified in Poplar^[17]^. PtoPHL3 binds to the promoter of *PtoPHT1s* (*PtoPHT1;3*, *PtoPHT1;4* and *PtoPHT1;5*) to enhance the Pi deficiency tolerance in poplar^[17]^. However, PtoWRKY40 can interact with PtoPHL3 and thus negatively regulate the expression of *PtoPHT1s*^[17]^. Notably, under Pi deficiency, the expression of *PtoWRKY40* is lower, and PtoPHL3 is free to activate the transcription of *PtoPHT1s* for PSR^[17]^. Twenty-one *PHR1/PHL* genes have been characterized in tea plants, while their function on PSR remains elusive^[138]^. Despite advances in the study of woody plant PHRs, many questions still need to be answered about the complete function of woody PHRs. For example, are the functions of woody plant PHRs consistent with those of herbaceous plants, do all woody plant PHRs necessarily respond to low phosphorus deficiencies, and do woody plants or gymnosperms have specific PHRs and perform functions unrelated to low phosphorus responses?

In citrus, *miR399* links Pi deficiency with the infection of huanglongbing (HLB), a devastating bacteria disease that is associated with '*Candidatus Liberibacter*' (Ca. L.)^[139]^. HLB-positive plants show a Pi deficiency symptom, and *miR399* is induced by HLB compared to healthy plants^[139]^. Supply of phosphorus can recover the Pi deficiency symptom of HLB in citrus^[139]^. *MiR399* also acts as a hub miRNA in regulating the sulfur and cadmium in poplar^[140]^. However, the roles of *miR399* and *miR827* directly involved in Pi deficiency signaling have not been characterized in woody plants. It is reported that other miRNAs are involved in Pi deficiency. *Pto-miRNA167*, *Pto-miRNA171*, *PtomiRNA394*, and *PtomiRNA857* are responses to low Pi and low nitrogen in poplar^[141]^.

## Interplay between Pi and other nutrients

Different mineral nutrients do not act independently^[150,151]^. Conversely, they depend highly on each other to achieve nutrient balance to maximize plant growth and productivity^[150]^. However, Pi also interacts with other mineral nutrients to regulate plant growth and development.

### Phosphorus-nitrogen interactions

**Figure 5:**
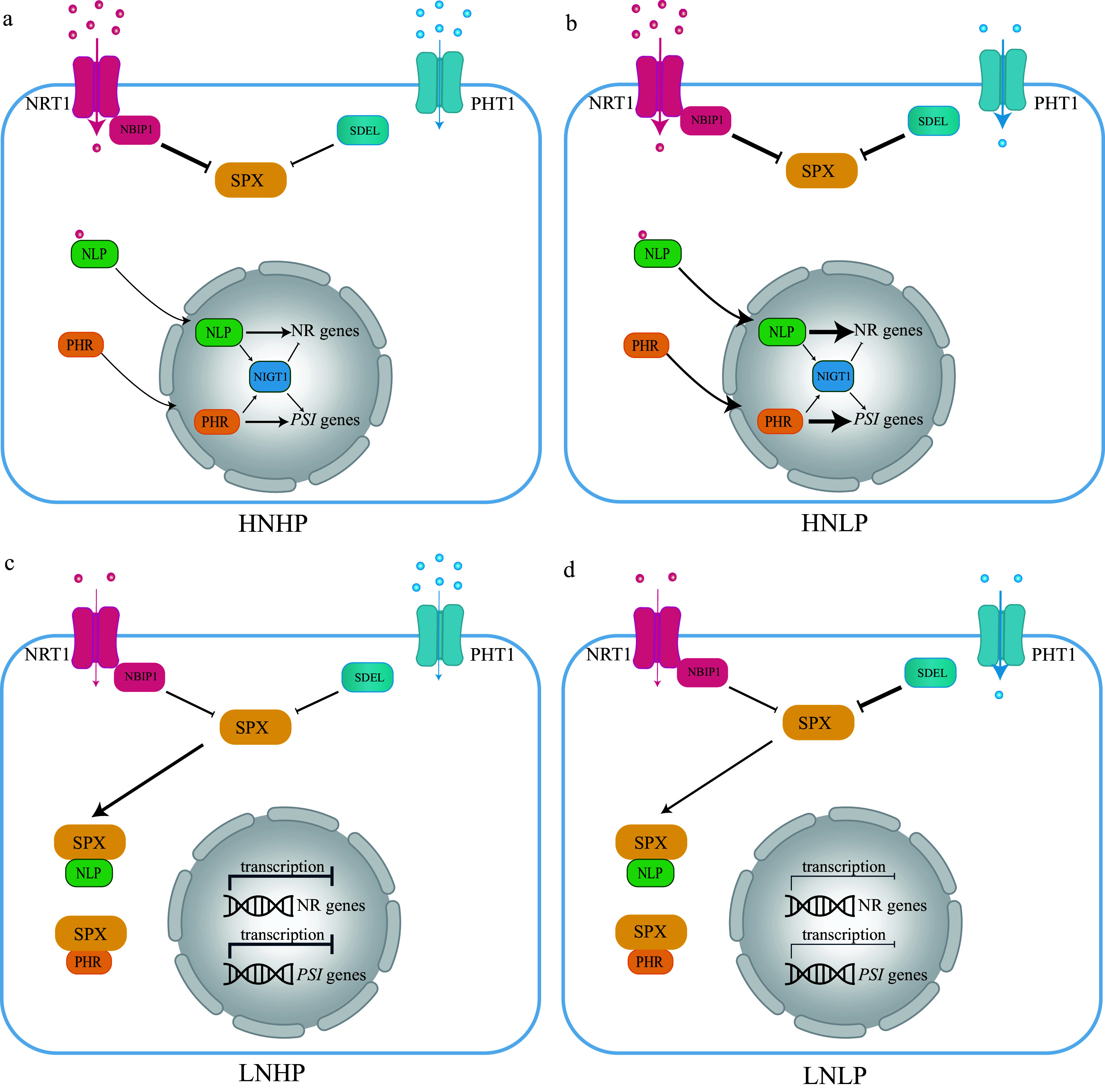
The integration network of nitrogen (N) and phosphorus (P) interactions based on Arabidopsis and rice. (a) Under high nitrate and high phosphate (HNHP) conditions, SPX protein, through NRT1.1-NBIP1, undergoes partial degradation and releases PHRs and NLPs from the cytoplasm into the nuclei to activate both PSI and nitrate-responsive gene expression. (b) Under high nitrate and low phosphate conditions (HNLP), NRT1,1-NBIP1 and SDELs mediate the degradation of SPX protein, leading to a significant reduction in SPX protein levels, thereby retaining PHR and NLP in the cytoplasm and repressing the expression of both PSI and nitrate-responsive genes. (c) and (d) SPX proteins tend to accumulate heavily in the cytoplasm in both high phosphate (HP) and low phosphate (LP) nitrate-limited environments (LNHP or LNLP). This can also lead to the retention of key regulatory factors like PHR1 and NLPs, and repression of the expression of both PSI and nitrate-responsive genes. Blue dot: Pi, Red dot: Nitrate.

Phosphorus and nitrogen are the two most indispensable mineral nutrients and a proper N:P ratio is important for plant growth^[150]^. Nitrate and Pi are the nutrient resources and act as signal molecules^[152]^. The nitrate transporter, Arabidopsis AtNRT1.1, and its rice homolog OsNRT1.1B are nitrate sensors to mediate the nitrate response^[153−156]^. In the canonical nitrate signaling pathway, NRT1.1 delivers the nitrate signaling by a Ca^2+^ dependent signaling cascade to regulate the key transcription factor Arabidopsis NIN Like Protein 7 (NLP7) and its rice homolog OsNLP3, and ultimately activate the transcription of nitrate response genes^[152,153,157]^. However, nitrate regulates nitrate and Pi signaling via the NRT1.1B-SPX4 module^[152]^ (Fig. 5a & b). This non-canonical nitrate signaling pathway triggers the OsNRT1.1B interaction and activates a ubiquitin E3 ligase-NRT1.1B Interacting Proteins (NBIPs)^[152]^. The NBIPs then trigger the ubiquitination of SPX4 and degrade SPX4^[152]^ (Fig. 5a & b). SPX4 interacts with NLP3 and PHR2 to inhibit the nitrate and low Pi signaling^[152]^ (Fig. 5a & b). Therefore, under nitrate-sufficient conditions, OsSPX4 is dissociated with NLP3 and PHR2, which leads to the simultaneous activation of the nitrate response and PSR^[152]^. In this way, the nitrate and Pi signaling are interplay and synergistic utilization to maximize plant growth and development^[152]^. Conversely, when nitrate is deficient, NRT1.1B is deactivated and NBIPs are not activated^[152]^ (Fig. 5c & d). SPX4 thus interacts with NLP3 and PHR2, leading to these two proteins localizing in the cytoplasm and blocking their transcription of nitrate response genes and PSR genes in the nucleus^[152]^ (Fig. 5c & d). The nitrate and Pi signaling are all disrupted^[152]^. It is also noteworthy that NIGT1, another transcription factor, the common downstream target of NLP and PHR, can simultaneously activate the nitrate response and PSR in Arabidopsis^[158−160]^.

The functions of NLP transcription factors on the nitrate signaling or nitrate-mediated lateral root development have been characterized in moso bamboo, and apple, respectively^[161,162]^. Although the phosphorus-nitrogen interaction remains unexplored in woody plants, one HD-ZIP transcription factor, PuHOX52, can simultaneously regulate the nitrate, Fe, and Pi responsive genes under N deficiency conditions in *Populus ussuriensis*^[163]^.

### Phosphorus-iron interactions

Phosphorus and iron have long been observed to antagonize each other^[149]^. Only in recent years has the molecular mechanism of phosphorus-iron antagonistic interaction gradually begun to be revealed^[149]^. The phosphorus-iron interplay has been described in the review, and it plays an essential role in the regulation of local Pi signaling. However, the phosphorus-iron interplay also regulates the PHR2-centered Pi homeostasis in rice^[149]^. Under Pi deficiency conditions, the Hemerythrin motif-containing Really interesting new gene and Zinc-finger protein 1 (HRZ1) and HRZ2, the ubiquitin E3-ligases of PHR2, are not activated, and thus, PHR2 protein abundance is increased^[149]^. The loss of HRZs negatively represses PHR2, ultimately leading to the activation of the transcription of PHR2-centered PSR genes^[149]^. Notably, PHR2 also inhibits the expression of HRZs to alleviate HRZs-directed PHR2 degradation further^[149]^. Conversely, under Fe deficiency conditions, the HRZs are activated, and PHR2 are degraded by ubiquitination; the PHR2-induced PSR genes are thus not transcribed^[149]^. Instead, the Fe starvation response genes are activated by HRZ-mediated transcription factors^[149]^. Notably, under Pi and Fe dual deficiency conditions, the PHR2 and HRZs all have a higher expression to promote plant tolerance to these stresses^[149]^. However, the phosphorus-iron interaction in woody plants has not been characterized.

Due to space limitations, we only summarize the current understanding of interactions between P and N and Fe in this review. However, P do interact with K^[164,165]^, S (Sulfur)^[146,166−168]^, B^[169−173]^, and Si (Silicon)^[174−179]^.

## Conclusions

Unraveling the physiological and molecular mechanisms of Pi uptake and utilization in woody plants and ultimately generating the high PUE woody plants by molecular genetics are important ways to achieve sustainable forestry development. Therefore, this review highlights several potential directions for future studies of Pi signaling and high PUE breeding in woody plants. Understanding how the Pi signaling functions in the formation of woody-specific traits such as wood formation or seasonal growth is the foundation of Pi research in woody plants. Therefore, it is important first to establish the Pi regulatory network in woody plants. The next goal is to decipher and functionally validate which genes or mechanisms are potential candidates for high PUE engineering in woody plants. These potential candidates may be key regulators such as transcription factors, kinases, transporters, RNA binding proteins, or key mechanisms such as epigenetic modification, RNA processing, RNA modification, and protein modification in woody plants^[161,180−183]^. Once the candidate genes have been characterized, these genes can be used in two directions. The first direction is using these key high PUE genes as molecular markers to select the elite germplasm. Another direction is generating high PUE woody plants by future molecular breeding methods such as transgenic or CRIPSR-Cas9 or CRIPSR-Cas12a. Therefore, this review systematically summarizes the current status of Pi phenotype, uptake, transport, and signaling studies in woody plants.

## Author contributions

The authors confirm contribution to the paper as follows: study conception and design: Ma L, Lu Z, Guo M; literature collection: Fang X; figure preparation: Deng L, Guo M, Ma L; draft manuscript preparation: Ma L, Fang X, Yang D, Guo M, Lu Z, Zhang Y; manuscript revision: Lin Z, Zhou J, Ma X, Chen C. All authors read and approved the final manuscript.

## Data availability

Data sharing is not applicable to this article as no datasets were generated or analyzed during the current study.
